# Irbesartan ameliorates diabetic kidney injury in *db/db* mice by restoring circadian rhythm and cell cycle

**DOI:** 10.2478/jtim-2022-0049

**Published:** 2024-05-21

**Authors:** Hailing Zhao, Zhiguo Li, Meihua Yan, Liang Ma, Xi Dong, Xin Li, Haojun Zhang, Ping Li

**Affiliations:** Beijing Key Lab Immune-Mediated Inflammatory Diseases, Institute of Clinical Medical Sciences, China–Japan Friendship Hospital, Beijing 100029, China; The Hebei Key Lab for Organ Fibrosis, the Hebei Key Lab for Chronic Disease, School of Public Health, International Science and Technology Cooperation Base of Geriatric Medicine, North China University of Science and Technology, Tangshan 063210, Hebei Province, China; Clinical Laboratory, China–Japan Friendship Hospital, Beijing 10029, China

**Keywords:** irbesartan, diabetic kidney disease, gene microarray, circadian rhythm, cell cycle

## Abstract

**Background and Objectives:**

Irbesartan has been widely used in the clinical treatment of diabetic kidney disease (DKD). However, the molecular mechanism of its delay of DKD disease progression has not been fully elucidated. The aim of the present study was to investigate the mechanism of irbesartan in the treatment of DKD.

**Materials and Methods:**

C57BL/KsJ *db/db* mice were randomly divided into the model group and irbesartan-treated group. After treatment with irbesartan for 12 weeks, the effects on blood glucose, body weight, 24-h urinary albumin, and renal injuries were evaluated. Microarray was used to determine the differentially expressed genes (DEGs) in the renal cortex of mice. |Log FC| <0.5 and false discovery rate (FDR) <0.25 were set as the screening criteria. Kyoto Encyclopedia of Genes and Genomes (KEGG), gene ontology (GO), protein–protein interaction (PPI) network and modules, and microRNA (miRNA)-DEGs network analysis were applied to analyze the DEGs. Furthermore, quantitative real-time polymerase chain reaction (qRT-PCR) was used to validate the results of microarray.

**Results:**

The present study demonstrated irbesartan could significantly improve the renal function in *db/db* mice through decreasing 24-h urinary albumin and alleviating the pathological injury of kidney. Irbesartan may affect the expression of numerous kidney genes involved in circadian rhythm, cell cycle, micoRNAs in cancer, and PI3K–AKT signaling pathway. In the miRNA-DEGs network, miR-1970, miR-703, miR-466f, miR-5135, and miR-132-3p were the potential targets for irbesartan treatment. The validation test confirmed that key genes regulating circadian rhythm (*Arntl*, *Per3*, and *Dbp*) and cell cycle (*Prc1*, *Ccna2*, and *Ccnb2*) were restored in *db/db* mice on treatment with Irbesartan.

**Conclusion:**

Generally, irbesartan can effectively treat DKD by regulating the circadian rhythm and cell cycle. The DEGs and pathways identified in the study will provide new insights into the potential mechanisms of irbesartan in the treatment of DKD.

## Introduction

Diabetes mellitus (DM) accounts for more than 30% morbidity of diabetic kidney disease (DKD) patients, which has been regarded as a leading cause of kidney failure and end-stage renal disease worldwide.^[1]^ Efforts have been made to explore the pathogenesis of DKD and to develop new therapeutic strategies. However, there is still no effective intervention to prevent renal function decline in patients with DKD. Interestingly, although the incidence of diabetes is increasing, multiple studies have demonstrated that strict blood glucose control and the use of renin–angiotensin system (RAS) blockers cause a significant decline in the incidence of DKD.^[2]^ RAS blockers used in DKD not only reduce proteinuria, but also delay disease progression, which is only partly explained by their hemodynamic effects.^[3]^

The intrarenal activation of RAS plays a pivotal role in the pathophysiology of DKD and participates in its progression. As the main effector molecule produced by RAS, angiotensin II (Ang II) can activate Ang II type 1 receptors (AT1R) and type 2 receptors (AT2R), which are crucial for the development of DKD. The activation of AT1R, associated with vasoconstriction, sodium reabsorption, and growth promotion, leads to inflammation and the production of reactive oxygen species (ROS).^[4,5]^ Therefore, blocking Ang II with an AT1 antagonist has become one of the best clinical options in the treatment of renal disease. Irbesartan is widely used for the treatment of DKD and was shown to ameliorate progressive glomerulosclerosis in animal models and slow disease progression in DKD patients.^[6]^ However, the molecular mechanisms of how RAS inhibition is used to delay the progression of DKD disease have not been fully elucidated.

Several studies have highlighted the importance of gene expression profiling to elucidate the mechanisms of disease progression and drug therapy.^[7,8]^ To the best of our knowledge, there is no gene expression profile analysis of irbesartan in DKD treatment. In the present study, high-throughput microarray screening was used to identify the differentially expressed genes (DEGs) in the kidneys of DKD model mice with or without irbesartan treatment. Then, the DEGs were used for gene ontology (GO) analysis, Kyoto Encyclopedia of Genes and Genomes (KEGG) pathway analysis, gene set enrichment analyses, protein–protein interaction (PPI) network and modules, and microRNA (miRNA)-DEGs network analysis to identify the genes critically involved in the development of DKD, which were followed with validation of DEGs by quantitative real-time polymerase chain reaction (qRT-PCR). These results will provide new insights into the role of irbesartan in the treatment of DKD and guide the clinical use of irbesartan.

## Materials and methods

### Animal experimentation

All mice used in this study were purchased from the Laboratory Animal Center of Peking University (Beijing, China), including 8-week-old male C57BL/KsJ *db/db* (*n* = 12) and *db/m* mice (*n* = 6), of which *db/m* mice were used as the control group. Mice were housed in a holding room with a 12-h light–dark cycle using controlled temperature (23°C ± 3°C) and humidity (55% ± 15%), with a free access to standard laboratory food and water. The *db/db* mice were randomly divided into the model group (*n* = 6) and irbesartan (Ambovi, Sanofi Winthrop Industrie)-treated group (*n* = 6). The *db/db* mice in the model group and treatment group were administered saline and irbesartan (225 mg/kg/day) by intragastric gavage for 12 weeks, respectively. At the end of the experiment, all the mice were sacrificed and blood and kidney tissues were collected for further analysis.

### Ethical approval

All study protocols have been reviewed and approved by the ethics committee of the China–Japan Friendship Hospital and were conducted in accordance with the guidelines of the National Institutes of Health on the Care and Use of Experimental Animals.

### Urinary albumin excretion and renal histology

Mice were placed in metabolic cages every 4 weeks to collect urine for 24 h and to measure the urine output. Urine albumin in mice was analyzed by enzyme-linked immunosorbent assay (ELISA) according to the manufacturer’s instructions (Bethyl Laboratories Inc., Montgomery, TX, USA). Kidney tissues were fixed with 10% neutral formalin. The fixed tissue was embedded in paraffin and 3-5 sections were cut, followed by staining with periodic acid-Schiff (PAS). The staining was observed under an optical microscope. The severity of glomerulosclerosis was assessed using the percentage of mesangial matrix, as we described previously.^[9]^

### RNA extraction, amplification, labeling, and hybridization

The renal cortex tissues of mice in each group were carefully collected and cryopreserved at -80°C for further analysis. Total RNA was extracted using TRIzol (Invitrogen, Carlsbad, CA, USA) according to the manufacturer’s instructions. Genomic DNA contamination was removed by incubating with DNase I at 37°C for 15 min. RNA purity and concentration were checked using the NanoPhotometer spectrophotometer (IMPLEN, CA, USA).

Samples with bright bands of ribosomal 28S to 18S RNAs in a ratio of >1.5 : 1 were used for microarray analysis. Microarray experiments were performed by CapitalBio Corporation (Beijing, China), a service provider authorized by Affymetrix, Inc. (Santa Clara, CA, USA), in accordance with the Affymetrix GeneChip manual. As much as 100 ng of total RNA was used for complementary DNA (cDNA) synthesis. Biotin-tagged cRNA was produced using the GeneChip IVT Labeling Kit (Affymetrix). Subsequently, 15 μg of fragmented cRNA, with Control Oligo B2 and eukaryotic hybridization controls (bioB, bioC, bioD, cre), was hybridized to the Affymetrix Mouse Genome 430 2.0 Array at 45°C for 16 h (Affymetrix GeneChip Hybridization Oven 640). After hybridization, the GeneChip arrays were washed and then stained with streptavidin phycoerythrinonan with Affymetrix GeneChip Fluidics Station 450, followed by scanning with Affymetrix GeneChip Scanner 3000 7G. The microarray data have been submitted to the Gene Expression Omnibus repository and are accessible through accession number GSE178939.

### Identification of DEGs

Data were analyzed using Affymetrix Expression Console and Transcriptome Analysis Console (TAC) software (Affymetrix). The gene array was run in triplicate, and the significance of the difference for each gene was determined by one-way analysis of variance (ANOVA). Differentially regulated genes were defined according to the threshold of |Log FC| <0.5 and a false discovery rate (FDR) <0.25.

### Functional and pathway enrichment analyses

Database for Annotation, Visualization, and Integrated Discovery (DAVID, http://david. ncifcrf.gov/) is an online tool that can investigate the biological meaning behind a mass of genes. In order to analyze DEGs at the functional level, GO and KEGG pathway enrichment analysis were performed. Adjusted *P* value <0.05 was set as the cutoff criterion.

### PPI network construction and module analysis

To explore the interactive relationship among DEGs, we submitted the DEGs to the Search Tool for the Retrieval of Interacting Genes (STRING) database with the confidence score >0.4 set as the cutoff criterion. Then, the PPI network was visualized using Cytoscape. Molecular Complex Detection (MCODE) was used to screen the modules of PPI network with the following criteria: K-core = 2, max. depth = 100, mode score cutoff = 0.2, and degree cutoff = 2. The function and pathway enrichment analyses in the module were performed by DAVID.

### miRNA-DEGs network analysis

The Mirwalk2 database (http://www.zmf.umm.uniheidelberg.de/apps/zmf/miRWalk2) provides maximum predictive and experimentally validated miR–target interactions with various miRNA databases. In this study, the Mirwalk2 database was used to predict miRNAs associated with DEGs. Finally, Cytoscape software 2.8 (www.cytoscape.org/; National Resource for Network Biology [NRNB], Bethesda, MD, USA) was used to construct and visualize miRNA–target gene interaction networks.

### Validation of DEGs by quantitative polymerase chain reaction

The expression levels of DEGs related with circadian rhythm and cell cycle were measured by quantitative polymerase chain reaction (qPCR) in triplicate for the technical validation of microarray data. Real-time quantitative analysis was carried out using renal RNA extracted from *db/m* mice (*n* = 3), *db/db* mice (*n* = 3), and *db/db* mice treated with irbesartan (*n* = 3), which was performed in three replicates. Results were expressed as fold expression relative to the expression in the control group by using the delta-delta Ct (ΔΔCt) method, and the control group in the study is *db/m* mice. The level of *Gapdh* RNA was used as an internal standard. All of these primers are listed in Table 1.

**Table 1:** List ands sequence of primers

Gene	Primer sequences
m*Arntl*-forward	TCAAGACGACATAGGACACCT
m*Arntl*-reverse	GGACATTGGCTAAAACAACAGTG
m*Per3*-forward	AAAAGCACCACGGATACTGGC
m*Per3*-reverse	GGGAGGCTGTAGCTTGTCA
m*Ccna2*-forward	GCCTTCACCATTCATGTGGAT
m*Ccna2*-reverse	TTGCTGCGGGTAAAGAGACAG
m*Ccnb*-forward	GCCAAGAGCCATGTGACTATC
m*Ccnb*-reverse	CAGAGCTGGTACTTTGGTGTTC
m*Prc1*-forward	CAGATGAGTCTATCACATGCCTG
m*Prc1*-reverse	CCTCGGTTCTTTGTAGCCTCT
m*Dbp*-forward	GGAAACAGCAAGCCCAAAGAA
m*Dbp*-reverse	CAGCGGCGCAAAAAGACTC
m*Gapdh*-forward	TGTTTCCTCGTCCCGTAGA
m*Gapdh*-reverse	ATCTCCACTTTGCCACTGC

### Statistical analysis

The mRNA expression levels of DEGs were determined using Statistical Package for the Social Sciences (SPSS) 21.0. The data are presented as means ± standard error of the mean (SEM), and independent-samples *t*-test was applied for the statistical analysis. *P* < 0.05 was accepted as statistically significant.

## Results

### Irbesartan alleviated proteinuria and athological changes in kidney

**Figure 1:**
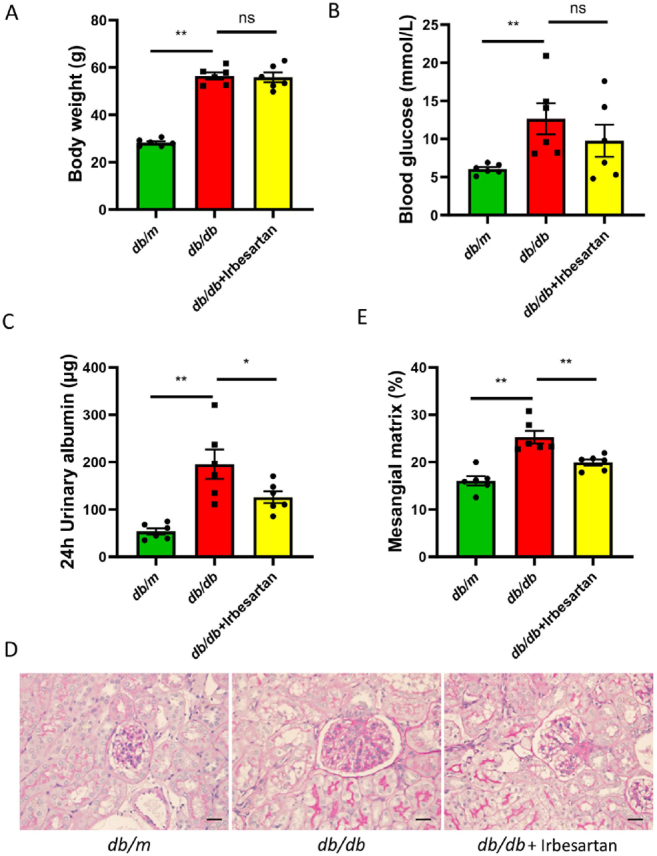
Irbesartan alleviates proteinuria and pathological injuries of kidney in *db/db* mice. (A and B) Body weight and blood glucose were measured at the end of the experiment. (C) Twenty-four-hour urinary albumin was found. The magnification of the images is 400×. (D) PAS staining was used to evaluate the renal injuries (scale bar, 50 μm). (E) Semi-quantitative measurements of mesangial matrix area were performed using PAS staining. Data are presented as the mean ± SEM. ^*^P < 0.05, ^**^P < 0.01. PAS: periodic acid-Schiff, SEM: standard error of the mean.

At the end of the experiment, we detected blood glucose, weight, and urinary albumin excretion, which were markedly increased in *db/db* mice compared to *db/m* mice. After 16 weeks of treatment with irbesartan, only urinary albumin excretion showed significantly lower values in the irbesartan treatment group than that in *db/db* mice (Figure 1A–C), indicating that irbesartan significantly improved the renal function in *db/db* mice.

Consistent with the proteinuria results, greater mesangial matrix dilation and thicker glomerular basement membrane of kidney were observed in *db/db* mice than in *db/m* mice, and these histological injuries were significantly ameliorated in *db/db* mice treated with irbesartan, as shown in Figure 1D and E.

### Microarray data analysis

**Figure 2:**
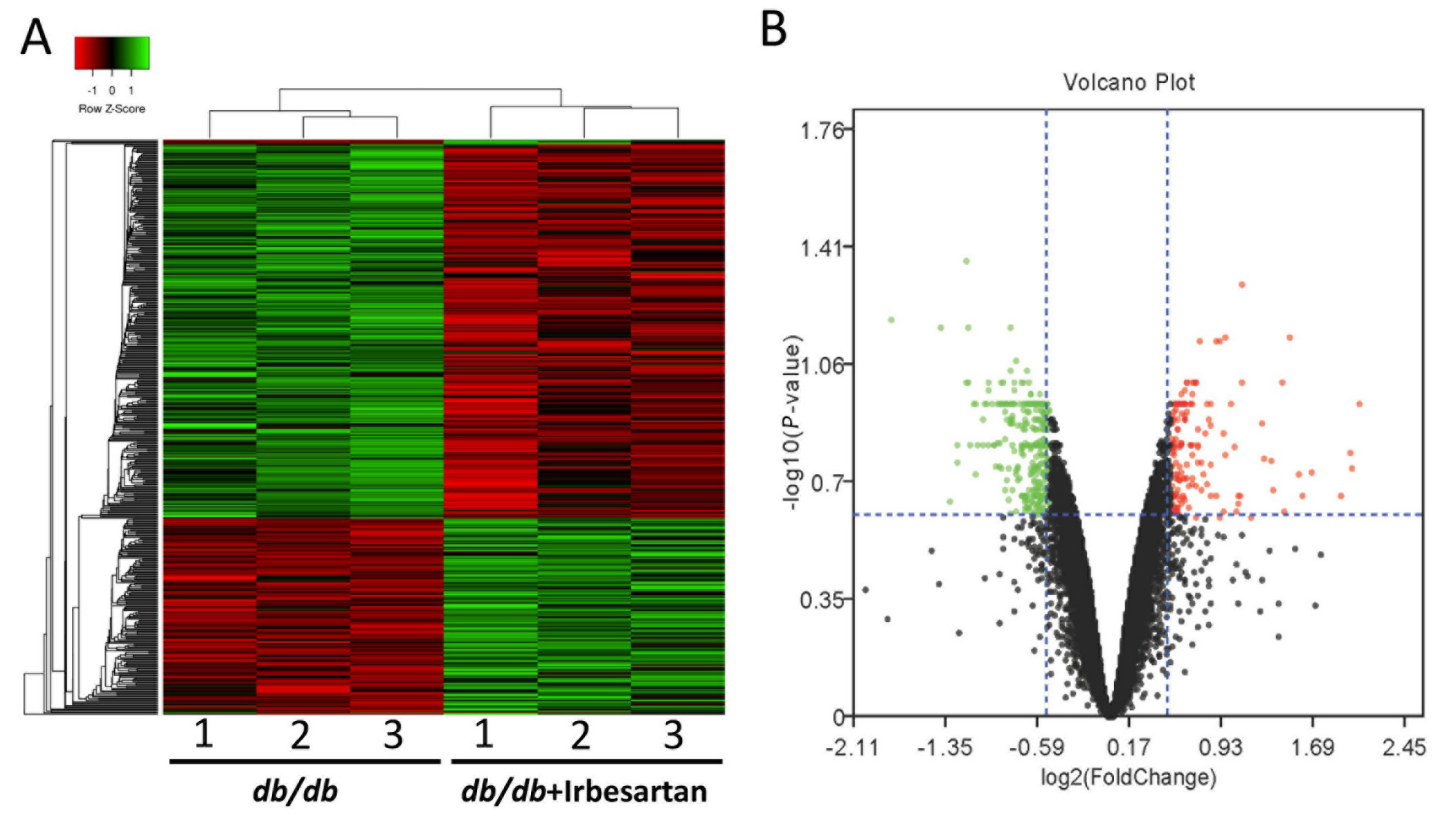
Identification of DEGs in kidneys of *db/db* mice with or without irbesartan treatment. (A) Heat map of DEGs between *db/db* mice and db/db mice + irbesartan. (B) Volcano plot shows the distribution of DEGs. DEGs: differentially expressed genes.

To explore the pharmacological target of irbesartan for T2DM kidney injury, three mice in each group were selected for renal mRNA expression profile analysis and the DEGs were analyzed. Only genes with |Log FC| <0.5 and FDR <0.25 were considered as DEGs. A total of 472 DEGs were identified, including 300 upregulated genes and 172 downregulated genes in *db/db* mice with irbesartan treatment, compared to untreated *db/db* mice. The top 30 genes with the most significant differences are listed in Table 2. DEGs were selected to generate the clustering heat map according to the fold change, and the clustering analysis of DEGs could well distinguish the *db/db* mice with or without irbesartan treatment (Figure 2A, B).

**Table 2:** Top 30 differentially expressed genes between *db/db* mice with and without irbesartan

Genes	logFC	*P*. adj	*P*-value
*Ciart*	1.99	0.01748	8.70E-07
*Ypel2*	-1.19	0.042906	4.27E-06
*Gm25279*	1.09	0.050326	7.51E-06
*Arntl*	-1.81	0.06402	1.27E-05
*Nfil3*	-1.40	0.067422	2.10E-05
*Gm26226*	-0.83	0.067422	2.28E-05
*Gm25401*	-1.18	0.067422	2.35E-05
*Plk1*	0.95	0.071817	3.10E-05
*Dbp*	1.48	0.071817	3.22E-05
*Lama1*	0.87	0.07415	4.08E-05
*Cdh11*	0.74	0.07415	4.40E-05
*Ldlr*	0.90	0.07415	4.43E-05
*Ren1*	-0.77	0.085439	5.53E-05
*Gm5779*	-0.82	0.091245	6.74E-05
*Rasl11a*	-0.69	0.091245	6.81E-05
*Slc16a1*	-1.19	0.098574	8.80E-05
*Slc25a30*	-0.68	0.098574	9.11E-05
*Rorc*	-0.80	0.098574	9.18E-05
*Wsb1*	-1.00	0.098574	9.63E-05
*Defb19*	0.71	0.098574	0.0001
*Zbtb16*	-1.17	0.098574	0.000106
*Nr1d1*	1.43	0.098574	0.000114
*Gm25383*	-0.73	0.098574	0.000116
*Gm25099*	-0.89	0.098574	0.000127
*Gm7241*	-0.72	0.098574	0.000133
*Ccnb2*	1.08	0.098574	0.000135
*Gm8765*	0.68	0.098574	0.000142
*Stard4*	0.63	0.098574	0.000143
*Hes6*	0.63	0.098574	0.000145
*Narf*	0.70	0.098574	0.000147

FC: fold change; *P*. adj: adjusted *P*-value.

### Functional enrichment analysis and KEGG pathway analysis

**Figure 3:**
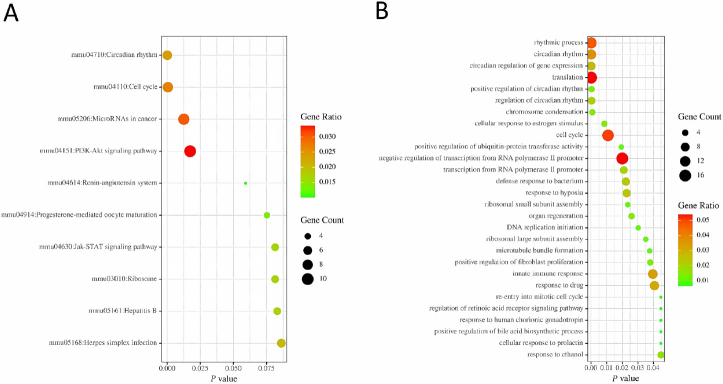
GO classification and KEGG pathway analysis of related DEGs. (A) GO enrichment analysis of DEGs in the biological process. (B) KEGG pathway analysis of DEGs. DEGs: differentially expressed genes, GO: gene ontology, KEGG: Kyoto Encyclopedia of Genes and Genomes.

To further investigate the biological functions of the DEGs, we performed GO and pathway analysis using DAVID. The results showed that DEGs were involved in several biological process (BP) and KEGG pathways. In the BP ontology, the DEGs were mainly enriched in rhythmic process (GO:0048511), circadian rhythm (GO:0007623), circadian regulation of gene expression (GO:0032922), regulation of circadian rhythm (GO:0042752), and positive regulation of circadian rhythm (GO:0042753) (Figure 3A). To gain further insight into the function of the identified DEGs, KEGG analysis identified four enriched pathways of the DEGs, including circadian rhythm (mmu04710), cell cycle (mmu04110), PI3K-AKT signaling pathway (mmu05206), and miRNAs in cancer (Figure 3B).

### PPI network construction and module analysis

**Figure 4:**
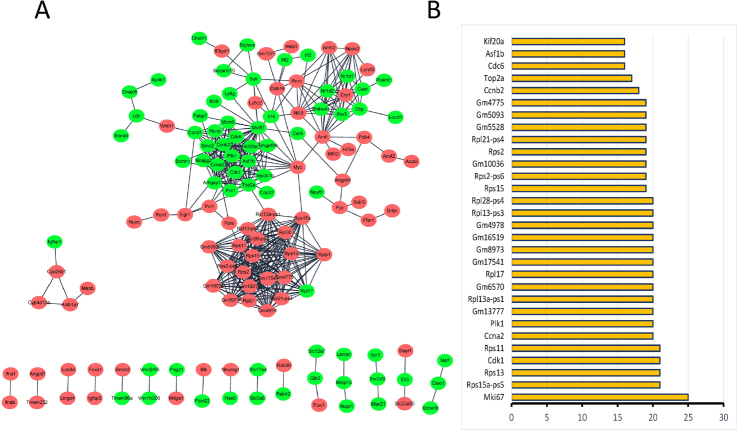
PPI network constructed with the DEGs to screen crucial genes. (A) PPI network for DEGs between *db/db* mice with and without irbesartan. Circles represent nodes and lines between nodes represent edges, which indicate DEGs and interactions between two DEGs, respectively. The red circle is the upregulated gene, and the green circle is the downregulated gene. (B) The most significant 30 node degree genes in the PPI network. DEGs: differentially expressed genes, PPI: protein–protein interaction.

**Figure 5:**
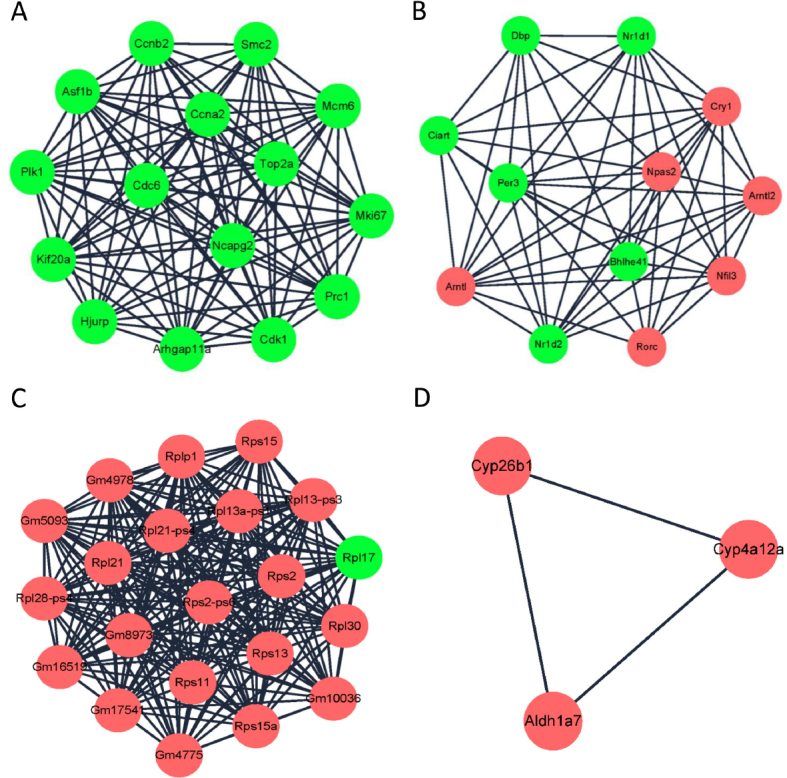
Significant modules screened from PPI network. Red circles represent upregulated genes and green ones represent downregulated genes in *db/db* mice treated with irbesartan. (A) Significant module is related with cell cycle. (B) Significant module is related with circadian rhythm. (C) Significant module is related with ribosome. (D) Significant module is related with drug metabolism. PPI: protein–protein interaction.

To evaluate the associations between the identified DEGs, a PPI network constructed with the DEGs consisted of 3844 nodes and 6727 edges (Figure 4A). Based on information from the STRING database, the DEGs with higher degrees (degree ≥20) were screened and selected as hub genes, including *Mki67, Rps15a-ps5, Rps13, Cdk1, Rps11, Ccna2, Plk1, Gm13777, Rps13a-ps1, Gm6570, Rpl17, Gm17541, Gm8973, Gm16519, Gm4978, Rpl13-ps3*, and *Rpl28-ps4* (Figure 4B). In addition, the top four most significant modules were selected, and module 1 was mainly enriched in cell cycle. Module 2 was mainly enriched in circadian rhythm. Furthermore, module 3, screened from the PPI network, was enriched in ribosome. Module 4 was enriched in drug metabolism (Figure 5A–D and Table 3).

**Table 3:** Significantly enriched functions for differentially expressed genes of the PPI network

Term ID	Term description	Gene count	FDR	Genes
GO:0007623	Circadian rhythm	12	0.0012	*Cry1, Rorc, Bhlhe41, Arntl, Npas2, Nfil3, Nr1d1, Klf10, Dbp, Per3, Arntl2, Ciart*
GO:0032922	Circadian regulation of gene expression	8	0.0013	*Cry1, Rorc, Bhlhe41, Arntl, Npas2, Nr1d1, Per3, Ciart*
GO:0042752	Regulation of circadian rhythm	10	0.0013	*Cry1, Rorc, Arntl, Npas2, Top2a, Nr1d1, Klf10, Nr1d2, Per3, Arntl2*
GO:0048511	Rhythmic process	15	0.0013	*Cry1, Rorc, Bhlhe41, Arntl, Npas2, Nfil3, Top2a, Nr1d1, Egr1, Klf10, Dbp, Nr1d2, Per3, Arntl2, Ciart*
mmu04710	Circadian rhythm	7	2.55E-05	*Cry1, Rorc, Bhlhe41, Arntl, Npas2, Nr1d1, Per3*
mmu03010	Ribosome	9	0.002	*Rps11, Rplp1, Rpl21, Rps15, Rpl17, Rps2, Rpl30, Rps15a, Rps13*
mmu04110	Cell cycle	8	0.006	*Cdk1, Myc, Mcm6, Ccna2, Plk1, Ccnb2, Cdc6, Ccnd1*

FDR: false discovery rate; PPI: protein–protein interaction.

### DEGs-related miRNAs

**Figure 6:**
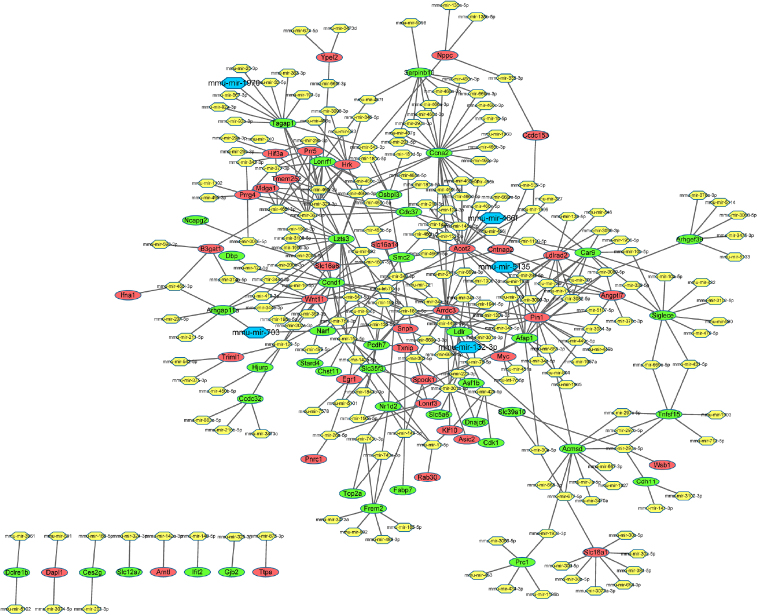
The miRNA–gene interaction network. Red ellipses indicate upregulated genes, and green ellipses indicate downregulated genes. Blue hexagons indicate the identified differential miRNAs from the DEGs. DEGs: differentially expressed genes, miRNA: microRNA.

After searching the Mirwalk2 database, we used 222 miRNAs that regulated the above DEGs to construct a miRNA-DEGs network, including 432 interaction pairs and 24 DEGs. Among them, mmu-miR-1970, mmu-miR-703, mmu-miR-466f, mmu-miR-5135, and miR-132-3p were identified to be the differentiated miRNAs according to DEGs from our microarray data, as shown in Figure 6. *Tagap1* and *Narf* were regulated by mmu-miR-1970; *Ldlr* was regulated by miR-132-3p; *Acot2* was regulated by mmu-miR-466f; and *Acot2* and *Ldlrad2* were regulated by mmu-miR-5135. The results indicate that irbesartan may regulate DEGs by regulating miRNA to improve DKD.

### Validation of hub genes in kidneys of db/db mice with or without irbesartan treatment

**Figure 7:**
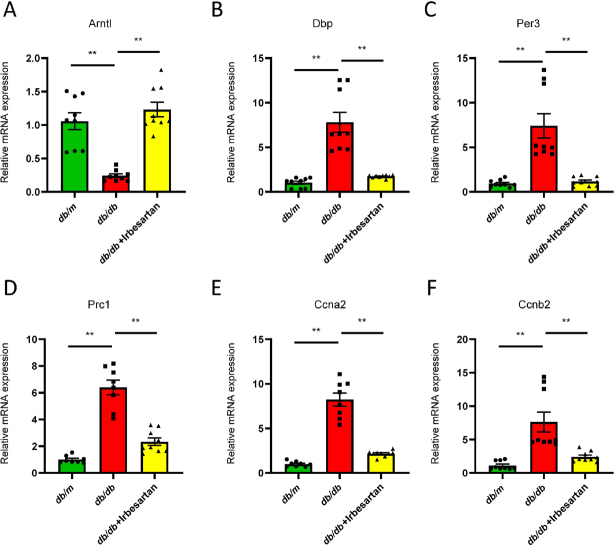
The differentially expressed genes validated by qRT-PCR. (A–C) The expression of circadian rhythm-related genes, including *Arntl*, *Dbp*, and *Per3*. (D, E) The expression of cell cycle-related genes, including *Prc1*, *Ccna2*, and *Ccnb2*. Data are expressed as the mean ± SEM, ^**^P < 0.01. qRT-PCR: quantitative real-time polymerase chain reaction, SEM: standard error of the mean.

According to the PPI network analysis, we verified the mRNA expression of representative genes in two pathways: circadian rhythm and cell cycle. As shown in Figure 7, significant upregulation of *Per3* and *Dbp* genes and significant downregulation of *Arntl* gene were confirmed in the kidney of *db/db* mice, which was significantly reversed in irbesartan-treated *db/db* mice (Figure 7A–C). In addition, the upregulated genes related to cell cycle in *db/db* mice, *Prc1*, *Ccna2*, and *Ccnb2*, were significantly downregulated by irbesartan (Figure 7D–E). These outcomes were consistent with the microarray results, demonstrating that circadian rhythm and cell cycle may be particularly important mechanisms in the treatment of DKD by irbesartan.

## Discussion

DKD is a major public health problem that harms people’s lives and health, and the main clinical features of DKD are proteinuria and a progressive decline in renal function, which are associated with structural and functional changes in the kidney.^[10]^ The pathogenesis of DKD is multifactorial, including genetic, metabolic, and hemodynamic factors.^[11]^ ARBs can effectively reduce urinary protein and delay the decline of renal function, so they are recommended as the first-line treatment of DKD.^[12]^ There is increasing evidence showing that irbesartan plays a key role in the treatment of DKD, which may be due to blocking the effects of Ang II on blood pressure, renal hemodynamic and nonhemodynamic effects, thereby inhibiting the growth-promoting, profibrotic, and other actions.^[13,14]^ Previous studies have also shown that irbesartan ameliorates DKD by suppressing the RANKL–RANK–NF-κB pathway in type 2 diabetic *db/db* mice.^[15]^ However, the protective mechanism of irbesartan on kidney may be complicated. Microarray gene expression profile is a high-throughput technique that can quickly generate the gene expression pattern of the sample at a given time point, and bioinformatics analysis helps to identify potential DEGs. Therefore, we used microarray gene expression profiles to explore the key genes and pathways of irbesartan in the treatment of DKD, so as to further improve the molecular mechanism of irbesartan therapy for DKD.

In the present study, we observed the kidney injury of *db/m* mice and *db/db* mice with or without irbesartan treatment. We found that irbesartan significantly reduced urinary albumin secretion and ameliorated renal histological injuries. To elucidate the underlying molecular mechanisms, we performed microarray profile screening of DEGs in *db/db* mice with or without irbesartan treatment. Four hundred and seventy-two DEGs were identified, including 300 upregulated genes and 172 downregulated genes, in *db/db* mice with irbesartan treatment compared to *db/db* mice. Functional enrichment analysis and KEGG pathway analysis showed DEGs were mainly enriched in circadian rhythm, cell cycle, miRNAs in cancer, and PI3K–AKT signaling pathway. PPI network construction and module analysis were also performed with the DEGs, and several DEGs with higher degrees were identified, including cell cycle, circadian rhythm, ribosome, and drug metabolism. What is more, the results of qRT-PCR were consistent with the results of gene expression microarray, which indicated that specific DEGs might play a key role in the treatment of DKD with irbesartan.

To the best of our knowledge, this study is the first to conduct a high-throughput analysis of gene expression patterns in the kidney of *db/db* mice and compare it with irbesartan treatment. Analysis of significant enrichment of DEGs in the BP showed the DEGs were mainly enriched in rhythmic process, circadian rhythm, and circadian regulation of gene expression. Circadian rhythm also showed the most significant difference in KEGG analysis, suggesting that abnormal circadian rhythm plays a key role in the occurrence of DKD and may be the vital target for irbesartan treatment. Therefore, renal tissue of mice was used to verify and analyze the expression of three key genes related to the circadian rhythm, including *Arntl, Per3*, and *Dbp*. The results revealed that compared to control mice, the model mice had lower expression of *Arntl* and higher expression of *Per3* and *Dbp*. Importantly, when *db/db* mice were treated with irbesartan, the expression of *Arntl* was significantly increased and the expression of *Per3* and *Dbp* was markedly downregulated. It is widely accepted that circadian rhythm is essential for maintaining physiological functions and homeostasis.^[16,17]^ Circadian rhythm is a universal phenomenon that plays an important role in maintaining physiological processes and regulating the adaptability of the body to internal and external environments. Currently, it is widely believed that the generation and maintenance of circadian rhythm depends on the clock feedback regulation system, which is composed of clock genes (*BMAL1, CLOCK, CRY, PER*) and clock control genes (*Rev-ERBα, RORα, DBP, TEF*, and *HLF*). Mammalian major feedback loops are activated by heterodimeric transcriptional activation of Brain and Muscle ARNT-like 1 (BMAL1 or ARNTL) and Circadian Locomotor Output Cycles protein Kaput (CLOCK). CLOCK and BMAL1 form a heterodimer and combine with the E response element (E-box) in the upstream promoter region of target genes (*PER, CRY, Rev-ERBα*, and *RORα*) to initiate the transcription process. The accumulation of PER and CRY proteins negatively regulates transcription of CLOCK–BMAL1 heterodimers. Moreover, Rev-ERBα binds to *BMAL1* promoter to inhibit BMAL1 transcription, whereas RORα acts as a positive regulator to promote *BMAL1* transcription. In addition, *DBP, TEF, HLF*, and other CLOCK control genes are also direct target genes of BMAL1–CLOCK heterodimer and participate in the regulation of circadian rhythm.^[18]^ It has been reported that *BMAL1*-knockout mice developed circadian rhythm disorders and metabolic damage, including impaired glucose and fatty acid metabolism, which are potential factors that accelerate the development of DKD.^[19]^

Circadian rhythm disorders increase the risk of various diseases, including sleep disorders, metabolic diseases, and cancer.^[20,21,22]^ As far as we know, there are few studies on the biological rhythm of DKD. Several studies have shown that circadian rhythm is strongly associated with chronic kidney disease (CKD), suggesting that it may be a potential mechanism for DKD.^[23,24]^ Disruption of the circadian clock in animal models has been shown to lead to abnormal blood pressure and disruption of the circadian rhythm pattern of water and electrolyte excretion in urine.^[25]^ A disrupted circadian rhythm is associated with an increased risk of hypertension, kidney fibrosis, and kidney stones.^[26,27,28]^ In addition, the renal circadian clock may interfere with the pharmacokinetics and/or pharmacodynamics of various drugs and is, therefore, an important consideration during the treatment of some renal diseases.^[29,30]^ Temporal pharmacological studies have shown that duration of treatment affects blood pressure control. A clinical study of patients with CKD has shown that antihypertensive drugs taken at night are more effective in controlling the blood pressure than those taken in the morning, suggesting that taking antihypertensive drugs such as valsartan before bedtime can improve the blood pressure in patients with CKD.^[24,31]^ Irbesartan, an ARB, is widely used in blood pressure control. Previous studies have shown that taking irbesartan once a night has a greater therapeutic effect on blood pressure during sleep than in the morning, as it significantly promotes reduction in blood pressure during sleep.^[32]^ Circadian rhythm of blood pressure exists in most healthy people, and loss of circadian rhythm of blood pressure can lead to damage of target organs, such as stroke and left ventricular hypertrophy. Irbesartan not only has a good antihypertensive effect, but also can restore the circadian rhythm of blood pressure, which is an effective choice for reducing cardiovascular risk.^[33]^ The present study demonstrates that irbesartan may improve diabetic renal injuries by restoring the circadian rhythm.

Many studies have shown that excessive proliferation of renal cells can aggravate the progression of glomerular sclerosis and renal interstitial fibrosis.^[34,35]^ The proliferation of glomerular cells causes the accumulation of extracellular matrix (ECM), which eventually leads to glomerular sclerosis. Inhibition of glomerular cell proliferation significantly reduced ECM accumulation.^[36]^ Renal interstitial fibrosis is an important pathological change in the progression of DKD to end-stage renal failure, and excessive proliferation of fibroblast cells is the key factor leading to renal interstitial fibrosis.^[35]^ Therefore, inhibition of fibroblasts may slow down or even reverse renal interstitial fibrosis. In this study, we found that irbesartan can regulate the expression of genes related to cell cycle in the kidney of *db/db* mice, suggesting that irbesartan may improve DKD by regulating cell proliferation. The protein encoded by Prc1 is involved in cytokinesis and has been shown to be the substrate for a variety of cyclin-dependent kinases (CDKs), and it is present at high levels during the S and G2/M phases of mitosis, but decreases sharply when cells enter the G1 phase.^[37]^*Ccna2* encodes a highly conserved cyclin protein that promotes transition through G1/S and G2/M by activating CDK2.^[38]^ It has been found that G2/M arrest in renal tubular epithelial cells leads to renal fibrosis by activating JNK signals and upregulating the production of profibrotic cytokines. Renal fibrosis can be reversed by bypassing G2/M arrest with a p53 inhibitor, suggesting that the cell cycle is a key target for the treatment of CKD.^[39]^ It has been found that irbesartan regulates the cell cycle by antagonizing AT1R, thus inhibiting the proliferation of breast cancer cells.^[40]^ Studies have shown that hypertrophy is an important pathological feature of renal diseases such as diabetic nephropathy. Ang II induced renal tubular epithelial cell cycle arrest in G0/G1 phase, resulting in hypertrophy. It was found that cell cycle was significantly reversed after irbesartan treatment, to improve cell hypertrophy.^[41]^ Our results support the idea that irbesartan may reduce diabetic kidney injury by regulating the cell cycle; however, further mechanistic studies are required.

miRNAs are small noncoding RNAs that regulate gene expression by inducing mRNA degradation to block protein translation. Increasing research evidence shows that miRNAs play a key role in the development of DKD and are expected to become potential biomarkers for DKD. Expression of miRNAs has been analyzed in renal biopsy, urine/urinary exosomes, and total blood/plasma/serum.^[42]^ Multiple miRNAs, including miR-21-5p, miR-29a-3p, miR-126-3p, miR-192-5p, miR-214-3p, and miR-342-3p, were persistently dysregulated in patients with DKD. These miRNAs are associated with multiple pathways related to the pathogenesis of DKD, such as apoptosis, fibrosis, and accumulation of ECM. ^[43]^ Therefore, miRNAs play an important role in the treatment and clinical diagnosis of DKD. Experimental evidence showed that Ang II could significantly reduce the expression of miR-133a and miR-29b in cardiomyocytes and increase the expression of its target gene *Col1A1*, leading to myocardial fibrosis. Irbesartan effectively prevented the downregulation of miR-133a and miR-29b in cardiomyocytes and inhibited the expression of *Col1A1* and myocardial fibrosis.^[44]^ In order to explore whether the therapeutic effect of irbesartan on DKD involved miRNAs, we also predicted miRNAs related to key DEGs. The results suggested that there were five miRNAs with significant differences in our data, including miR-1970, miR-703, miR-466f, miR-5135, and miR-132-3p. In an expression profile of miRNA in the kidney of DKD model mice, it was found that the expression of miR-703 and miR-466f in the kidney of DKD mice was significantly increased, which is consistent with our results.^[45]^ Studies have found that miR-132-3p promotes cell proliferation by directly regulating *BCL2L11*, an apoptosis activator, leading to tuberous sclerosis.^[46]^ In addition, miR-132-3p could also promote oxidative stress by targeting *FOXO3*, leading to autosomal dominant polycystic kidney disease.^[47]^ However, there is no report on the above five miRNAs in DKD. Therefore, further experimental studies are needed to prove their role in the occurrence of DKD.

### Limitations

In the present study, we have demonstrated that irbesartan could effectively treat DKD by regulating the circadian rhythm and cell cycle in diabetic *db/db* mice. However, there are some limitations to our study. First, although the diabetic *db/db* mice model has the metabolic disorders characteristic of diabetic nephropathy, it is only an animal model reflecting early to middle-stage diabetic nephropathy, with relatively mild renal pathological damage, and therefore may have limited relevance to the actual clinical status. Second, our study showed that irbesartan was effective in reducing kidney injury in *db/db* mice, but a dosing effect analysis was performed. Moreover, the results confirmed that circadian rhythm and cell cycle played an important role in treatment of DKD with irbesartan; however, its potential regulatory mechanisms still need to be validated *in vivo* or *in vitro*.

## Conclusion

Our study suggests Irbesartan is an effective treatment for DKD, and its mechanism is related to the restoration of circadian rhythm and cell proliferation, and the key role of miRNA may be involved. These conclusions need to be confirmed by further mechanistic studies, and the potential mechanisms need to be further verified.

## References

[1] Umanath K, Lewis JB (2018). Update on Diabetic Nephropathy: Core Curriculum 2018. Am J Kidney Dis.

[2] Warren AM, Knudsen ST, Cooper ME (2019). Diabetic nephropathy: an insight into molecular mechanisms and emerging therapies. Expert Opin Ther Targets.

[3] Malek V, Suryavanshi SV, Sharma N, Kulkarni YA, Mulay SR, Gaikwad AB (2021). Potential of Renin-Angiotensin-Aldosterone System Modulations in Diabetic Kidney Disease: Old Players to New Hope!. Rev Physiol Biochem Pharmacol.

[4] Qin B, Wang Q, Lu Y, Li C, Hu H, Zhang J (2018). Losartan Ameliorates Calcium Oxalate-Induced Elevation of Stone-Related Proteins in Renal Tubular Cells by Inhibiting NADPH Oxidase and Oxidative Stress. Oxid Med Cell Longev.

[5] Panico K, Abrahao MV, Trentin-Sonoda M, Muzi-Filho H, Vieyra A, Carneiro-Ramos MS. (2019). Cardiac Inflammation after Ischemia-Reperfusion of the Kidney: Role of the Sympathetic Nervous System and the Renin-Angiotensin System. Cell Physiol Biochem.

[6] Koszegi S, Molnar A, Lenart L, Hodrea J, Balogh DB, Lakat T (2019). RAAS inhibitors directly reduce diabetes-induced renal fibrosis via growth factor inhibition. J Physiol.

[7] Ciccocioppo R, Panelli S, Conti Bellocchi MC, Cangemi GC, Frulloni L, Capelli E (2018). The Transcriptomic Analysis of Circulating Immune Cells in a Celiac Family Unveils Further Insights Into Disease Pathogenesis. Front Med (Lausanne).

[8] Zhao YB, Li W, Zhang Q, Yin Y, Yang CJ, Xu WX (2020). Distinct miRNA Gene Expression Profiles Among the Nodule Tissues of Lung Sarcoidosis, Tuberculous Lymphadenitis and Normal Healthy Control Individuals. Front Med (Lausanne).

[9] Zhang H, Zhao T, Gong Y, Dong X, Zhang W, Sun S (2014). Attenuation of diabetic nephropathy by Chaihuang-Yishen granule through anti-inflammatory mechanism in streptozotocin-induced rat model of diabetics. J Ethnopharmacol.

[10] Lin YC, Chang YH, Yang SY, Wu KD, Chu TS (2018). Update of pathophysiology and management of diabetic kidney disease. J Formos Med Assoc.

[11] Yoshida Y, Kashiwabara K, Hirakawa Y, Tanaka T, Noso S, Ikegami H (2020). Conditions, pathogenesis, and progression of diabetic kidney disease and early decliner in Japan. BMJ Open Diabetes Res Care.

[12] He D, Zhang Y, Zhang W, Xing Y, Guo Y, Wang F (2020). Effects of ACE Inhibitors and Angiotensin Receptor Blockers in Normotensive Patients with Diabetic Kidney Disease. Horm Metab Res.

[13] Simoes ESAC, Teixeira MM. (2016). ACE inhibition, ACE2 and angiotensin-(1-7) axis in kidney and cardiac inflammation and fibrosis. Pharmacol Res.

[14] Rodrigues Prestes TR, Rocha NP, Miranda AS, Teixeira AL, Simoes ESAC (2017). The Anti-Inflammatory Potential of ACE2/Angiotensin-(1-7)/ Mas Receptor Axis: Evidence from Basic and Clinical Research. Curr Drug Targets.

[15] Chen XW, Du XY, Wang YX, Wang JC, Liu WT, Chen WJ (2016). Irbesartan Ameliorates Diabetic Nephropathy by Suppressing the RANKL-RANK-NF-κB Pathway in Type 2 Diabetic db/db Mice. Mediators Inflamm.

[16] Huang W, Ramsey KM, Marcheva B, Bass J (2011). Circadian rhythms, sleep, and metabolism. J Clin Invest.

[17] Huang S, Jiao X, Lu D, Pei X, Qi D, Li Z (2020). Recent advances in modulators of circadian rhythms: an update and perspective. J Enzyme Inhib Med Chem.

[18] Olaoye OA, Masten SH, Mohandas R, Gumz ML (2019). Circadian Clock Genes in Diabetic Kidney Disease (DKD). Curr Diab Rep.

[19] Pan X, Mota S, Zhang B (2020). Circadian Clock Regulation on Lipid Metabolism and Metabolic Diseases. Adv Exp Med Biol.

[20] Faulkner SM, Bee PE, Meyer N, Dijk DJ, Drake RJ (2019). Light therapies to improve sleep in intrinsic circadian rhythm sleep disorders and neuropsychiatric illness: A systematic review and meta-analysis. Sleep Med Rev.

[21] Gabriel BM, Zierath JR (2019). Circadian rhythms and exercise - re-setting the clock in metabolic disease. Nat Rev Endocrinol.

[22] Maiese K (2017). Moving to the Rhythm with Clock (Circadian) Genes, Autophagy, mTOR, and SIRT1 in Degenerative Disease and Cancer. Curr Neurovasc Res.

[23] Zhang J, Rao J, Liu M, Zhou W, Li Y, Wu J (2020). Abnormal circadian rhythm of urinary sodium excretion correlates closely with hypertension and target organ damage in Chinese patients with CKD. Int J Med Sci.

[24] Ohashi N, Isobe S, Ishigaki S, Yasuda H (2017). Circadian rhythm of blood pressure and the renin-angiotensin system in the kidney. Hypertens Res.

[25] Hill AM, Crislip GR, Stowie A, Ellis I, Ramsey A, Castanon-Cervantes O (2021). Environmental circadian disruption suppresses rhythms in kidney function and accelerates excretion of renal injury markers in urine of male hypertensive rats. Am J Physiol Renal Physiol.

[26] Lin L, Zhang H, Yang J, Zhang J, Li K, Huo B (2016). Nocturnal and Circadian Rhythm of Blood Pressure Is Associated with Renal Structure Damage and Function in Patients with IgAN. Arch Med Res.

[27] Motohashi H, Tahara Y, Whittaker DS, Wang HB, Yamaji T, Wakui H (2020). The circadian clock is disrupted in mice with adenine-induced tubulointerstitial nephropathy. Kidney Int.

[28] Kushwaha RS, Gupta RC, Sharma JP, Sharma S, Singh RK, Cornelissen G (2017). Circadian Periodicity of Circulating Plasma Lipid Peroxides, Uric Acid and Ascorbic Acid in Renal Stone Formers. Indian J Clin Biochem.

[29] Torres R, Kramer WG, Baroldi P (2015). Pharmacokinetics of the dual melatonin receptor agonist tasimelteon in subjects with hepatic or renal impairment. J Clin Pharmacol.

[30] Firsov D, Bonny O (2018). Circadian rhythms and the kidney. Nat Rev Nephrol.

[31] Di Daniele N, Fegatelli DA, Rovella V, Castagnola V, Gabriele M, Scuteri A (2017). Circadian blood pressure patterns and blood pressure control in patients with chronic kidney disease. Atherosclerosis.

[32] Hermida RC, Ayala DE, Fernandez JR, Portaluppi F, Fabbian F, Smolensky MH (2011). Circadian rhythms in blood pressure regulation and optimization of hypertension treatment with ACE inhibitor and ARB medications. Am J Hypertens.

[33] Douma LG, Gumz ML (2018). Circadian clock-mediated regulation of blood pressure. Free Radic Biol Med.

[34] Masuda Y, Shimizu A, Kataoka M, Arai T, Ishikawa A, Du X (2010). Inhibition of capillary repair in proliferative glomerulonephritis results in persistent glomerular inflammation with glomerular sclerosis. Lab Invest.

[35] Li M, Jia F, Zhou H, Di J, Yang M (2018). Elevated aerobic glycolysis in renal tubular epithelial cells influences the proliferation and differentiation of podocytes and promotes renal interstitial fibrosis. Eur Rev Med Pharmacol Sci.

[36] Wang S, Wen X, Han XR, Wang YJ, Shen M, Fan SH (2018). Repression of microRNA-382 inhibits glomerular mesangial cell proliferation and extracellular matrix accumulation via FoxO1 in mice with diabetic nephropathy. Cell Prolif.

[37] Hernandez-Ortega S, Sanchez-Botet A, Quandt E, Masip N, Gasa L, Verde G (2019). Phosphoregulation of the oncogenic protein regulator of cytokinesis 1 (PRC1) by the atypical CDK16/CCNY complex. Exp Mol Med.

[38] Yan J, Hao C, DeLucia M, Swanson S, Florens L, Washburn MP (2015). CyclinA2-Cyclin-dependent Kinase Regulates SAMHD1 Protein Phosphohydrolase Domain. J Biol Chem.

[39] Yang L, Besschetnova TY, Brooks CR, Shah JV, Bonventre JV (2010). Epithelial cell cycle arrest in G2/M mediates kidney fibrosis after injury. Nat Med.

[40] Du N, Feng J, Hu LJ, Sun X, Sun HB, Zhao Y (2012). Angiotensin II receptor type 1 blockers suppress the cell proliferation effects of angiotensin II in breast cancer cells by inhibiting AT1R signaling. Oncol Rep.

[41] Liu BC, Sun J, Chen Q, Luo DD, Ma KL, Ruan XZ (2004). Effect of irbesartan on angiotensin II-induced hypertrophy of human proximal tubular cells. Chin Med J (Engl).

[42] Assmann TS, Recamonde-Mendoza M, de Souza BM, Bauer AC, Crispim D (2018). MicroRNAs and diabetic kidney disease: Systematic review and bioinformatic analysis. Mol Cell Endocrinol.

[43] Nascimento LRD, Domingueti CP (2019). MicroRNAs: new biomarkers and promising therapeutic targets for diabetic kidney disease. J Bras Nefrol.

[44] Castoldi G, Di Gioia CR, Bombardi C, Catalucci D, Corradi B, Gualazzi MG (2012). MiR-133a regulates collagen 1A1: potential role of miR-133a in myocardial fibrosis in angiotensin II-dependent hypertension. J Cell Physiol.

[45] Chen YQ, Wang XX, Yao XM, Zhang DL, Yang XF, Tian SF (2012). Abated microRNA-195 expression protected mesangial cells from apoptosis in early diabetic renal injury in mice. J Nephrol.

[46] Cai Y, Wang W, Guo H, Li H, Xiao Y, Zhang Y (2018). miR-9-5p, miR-124-3p, and miR-132-3p regulate BCL2L11 in tuberous sclerosis complex angiomyolipoma. Lab Invest.

[47] Choi S, Kim DY, Ahn Y, Lee EJ, Park JH (2021). Suppression of Foxo3-Gatm by miR-132-3p Accelerates Cyst Formation by Up-Regulating ROS in Autosomal Dominant Polycystic Kidney Disease. Biomol Ther (Seoul).

